# Supplementary material for: Mercury and Alzheimer’s Disease: Hg(II) Ions Display Specific Binding to the Amyloid-β Peptide and Hinder Its Fibrillization
Source: Biomolecules. 2019 Dec 27;10(1):44. doi: 10.3390/biom10010044 (PMC7022868; doi:10.3390/biom10010044)
Supplement: Supplementary file 1 [file biomolecules-10-00044-s001.pdf]

Supplementary Material

Mercury and Alzheimer's disease: Hg(II) ions display specific binding to the amyloid-β peptide and hinder its fibrillization

Cecilia Wallin[1], Merlin Friedemann[2], Sabrina B. Sholts[3], Andra Noormägi[2], Teodor Svantesson[1], Jüri Jarvet[1,4], Per M. Roos[5,6], Peep Palumaa[2], Astrid Gräslund[1], Sebastian K. T. S. Wärmländer[1,*]

[1] Department of Biochemistry and Biophysics, Stockholm University, Sweden.
[2] Department of Chemistry and Biotechnology, Tallinn University of Technology, Estonia.
[3] Department of Anthropology, National Museum of Natural History, Smithsonian Institution, Washington, DC, USA.
[4] The National Institute of Chemical Physics and Biophysics, Tallinn, Estonia.
[5] Institute of Environmental Medicine, Karolinska Institutet, Stockholm, Sweden.
[6] Department of Clinical Physiology, Capio St. Göran Hospital, Stockholm, Sweden.

* Correspondence: seb@dbb.su.se; Tel.: +46 8 162444

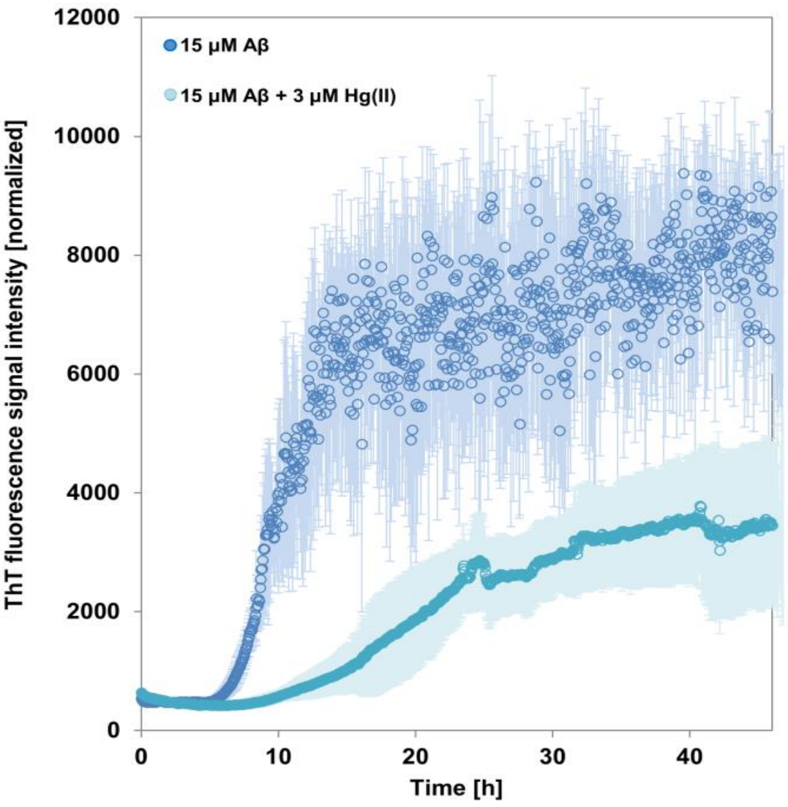

Figure S1. Amyloid fibril formation of Aβ40 peptides monitored by Thioflavin T (ThT) aggregation kinetics assay. ThT fluorescence signal intensity traces of averaged data from samples with 15 μM Aβ40 peptides in 20 mM sodium phosphate buffer pH 7.4 and 40 μM ThT incubated in the absence and presence of 3 μM Hg(II) ions at +37 °C under quiescent conditions. Standard deviations of four replicates are shown.

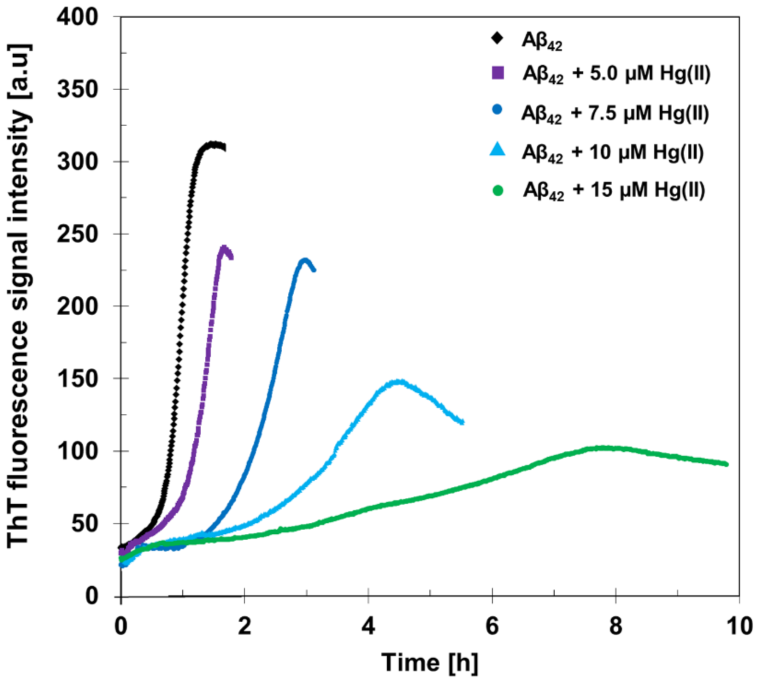

Figure S2. **Amyloid fibril formation of Aβ42 peptides monitored by Thioflavin T (ThT) aggregation kinetics assay.** ThT fluorescence signal intensity traces from samples with 5 µM Aβ42 peptides in 20 mM HEPES containing 100 mM NaCl pH 7.3 and 5 µM ThT, incubated in the absence and presence of 5-15 µM $Hg(NO_3)_2$ at +45 °C incubated with magnetic stirring. One series of Aβ42 peptides in the presence of increasing concentrations of Hg(II) ions is shown.

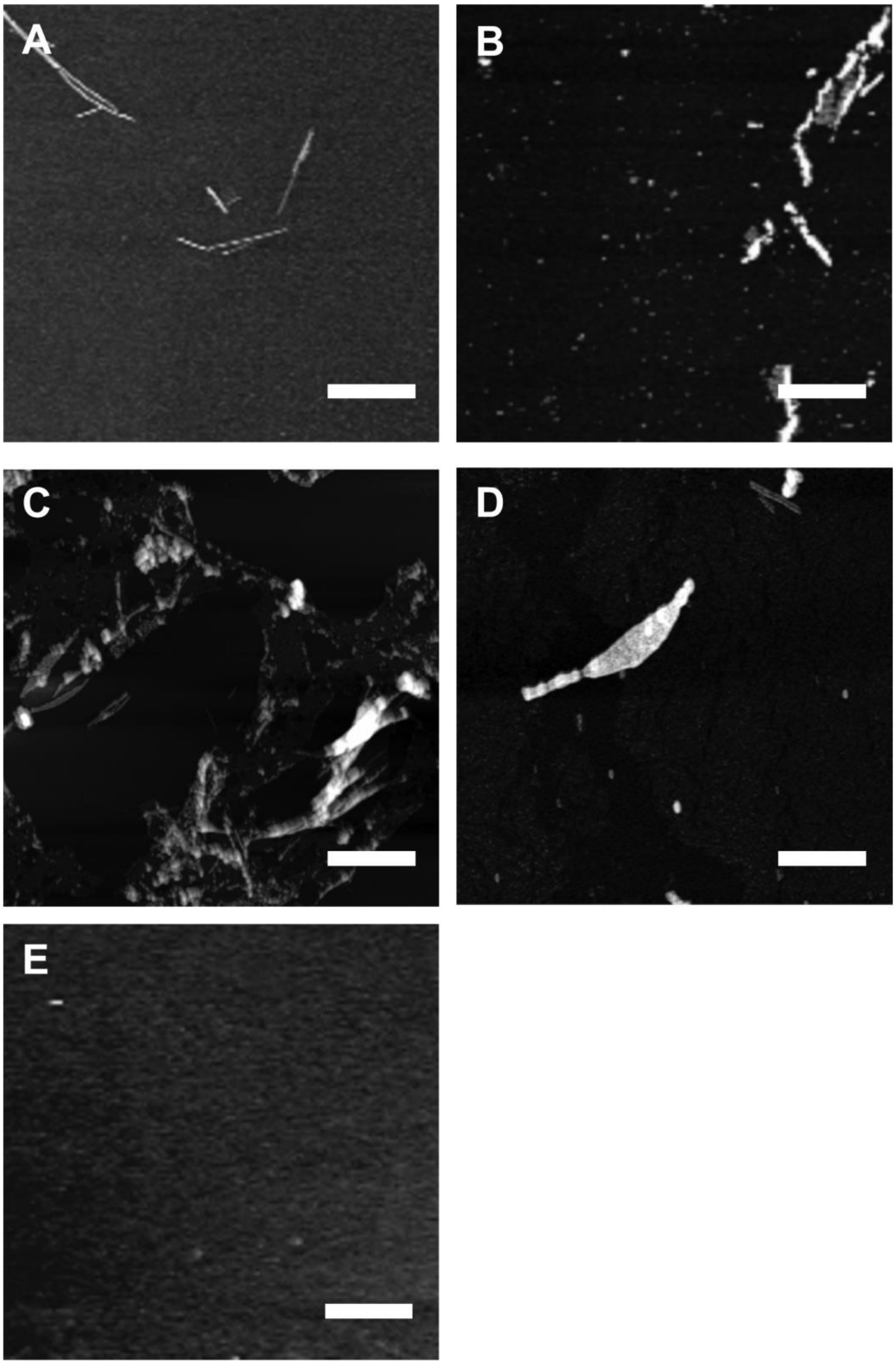

Figure S3. Solid state AFM imaging of fibril formation and morphology. (A-D) Topographical images of samples taken from the end of the ThT aggregation experiment (~45 h) in Figure 1. **(A)** shows 15 μM aggregated Aβ peptides alone, **(B)** Aβ + 0.8 μM Hg(II) ions, **(C)** Aβ + 1.5 μM Hg(II) ions, **(D)** Aβ + 3.0 μM Hg(II) ions, and **(E)** Aβ + 15 μM Hg(II) ions. The scale bars represent 1 μm.

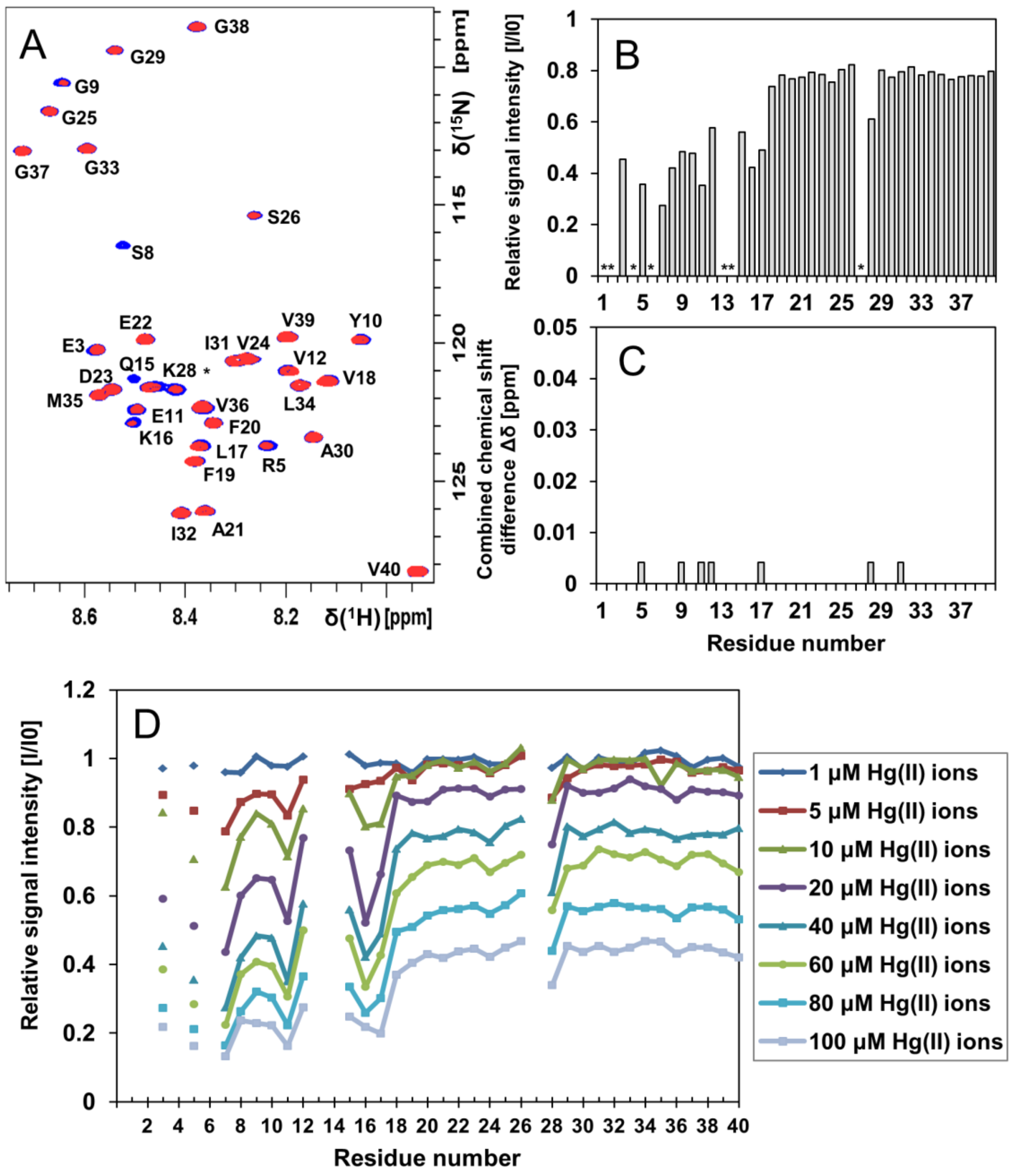

Figure S4. 2D NMR ^{1}H-^{15}N-HSQC titration experiments showing Aβ_{40} residue-specific perturbations from Hg(II) ions in buffer. (A) 700 MHz ^{1}H-^{15}N-HSQC spectra of 84 µM monomeric ^{13}C-^{15}N-labeled Aβ_{40} peptides alone (blue) and in the presence of 40 µM Hg(II) ions (red) in 20 mM sodium phosphate buffer pH 7.35 at +5 °C. In **(B)**, relative signal intensities determined from the amplitude of the amide crosspeaks in the two spectra in **(A)** are shown. Combined chemical shift differences from the spectra in **(A)** are shown in **(C)**. The relative signal intensities determined from the amplitude of the amide crosspeaks for the whole Hg(II) ions titration series are shown in **(D)**. Residues assigned with a * are not accurately determined or observed because of too fast exchange with the solvent or due to spectral overlap.

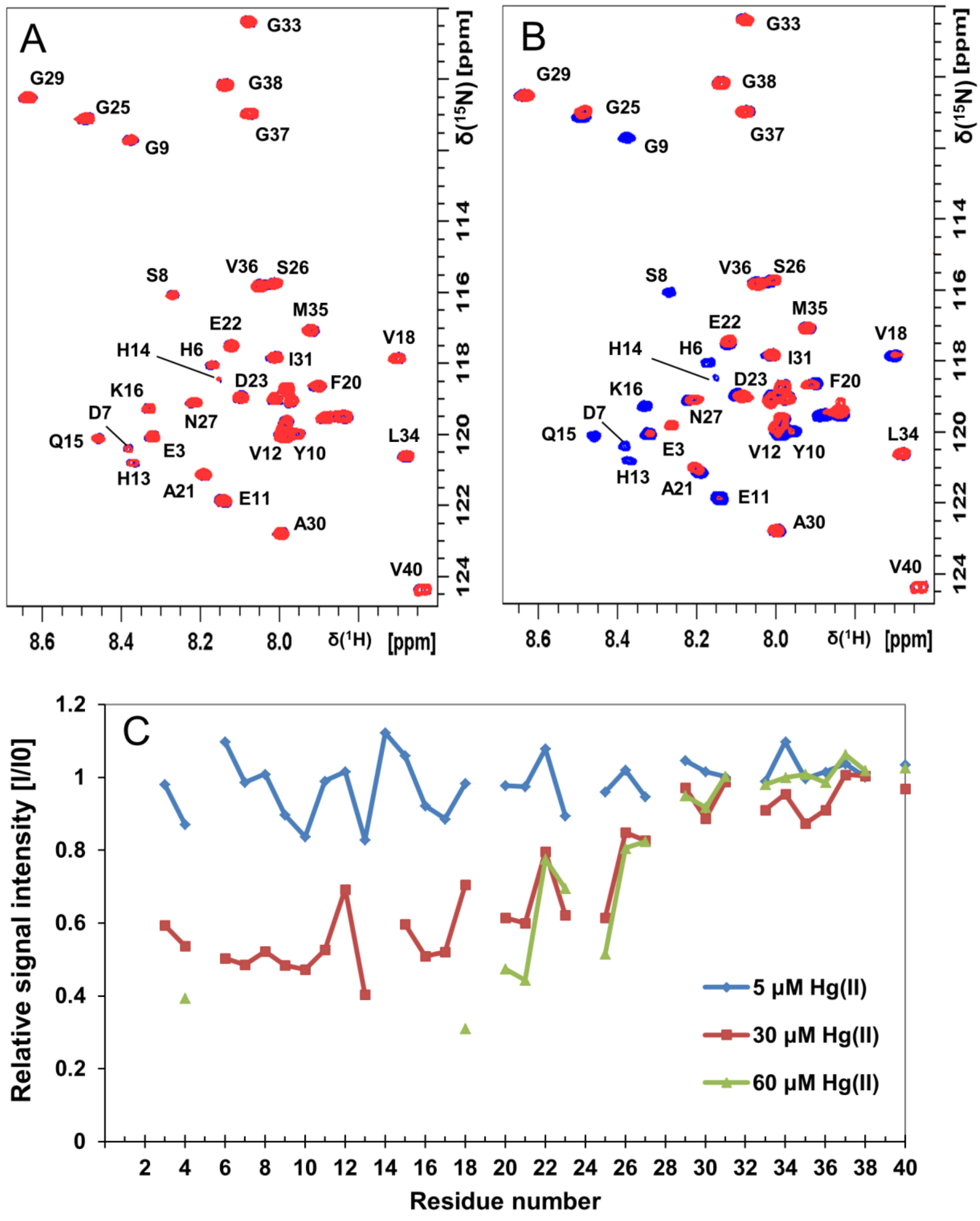

Figure S5. 2D NMR ^{1}H-^{15}N-HSQC titration experiments showing Aβ$_{40}$ residue-specific perturbations from Hg(II) ions in SDS micelles. 500 MHz ^{1}H-^{15}N-HSQC spectra of 84 µM monomeric ^{15}N-labeled Aβ$_{40}$ peptides (blue) in 20 mM sodium phosphate buffer pH 7.35 and 50 mM SDS at +25 °C. Red amide crosspeaks corresponds to 84 µM ^{15}N-labeled Aβ$_{40}$ peptides in the presence of 5 µM Hg(II) ions **(A)** or 60 µM Hg(II) ions **(B).** In **(C)** the relative signal intensities determined from the amplitude of the amide crosspeaks for the whole Hg(II) ions titration series are shown.
